# Energy and nutrient intake by 11–13-year-old young adolescents attending private schools in Delhi, India

**DOI:** 10.1017/S000711452400120X

**Published:** 2024-08-14

**Authors:** Anupama Ivaturi, Lynne Giles, Loc G. Do, Tina Rawal, Monika Arora, Paula Moynihan

**Affiliations:** 1 Adelaide Dental School, The University of Adelaide, Adelaide, SA, Australia; 2 School of Public Health, The University of Adelaide, Adelaide, SA, Australia; 3 School of Dentistry, University of Queensland, Herston, QLD, Australia; 4 Public Health Foundation of India, Gurgaon, Haryana, India

**Keywords:** Eating: Nutrients, Dietary fats, Dietary reference intakes, Nutrition status

## Abstract

There are no high-quality data on dietary behaviour of adolescents in India. This study aimed to assess the intake of energy (E), macronutrients and selected micronutrients in a sample of 11–13-year-old schoolchildren in Delhi, India. Participants from private schools (*n*=10) recorded dietary intake using a 3-d food diary. Information was entered into the dietary assessment tool, Intake24, to ascertain portion size and convert data into nutrient intake through integrated food tables. Of the 514 consenting participants, 393 (76·4 %) (169 girls, 224 boys) aged 11·4 (±1·8) years completed the study. The median (interquartile range (IQR) daily E intake was 2580 (2139·3–2989·8) kcal (10·8 (9·0 − 12·5) MJ) for girls and 2941·5 (2466·7–3599·3) kcal (12·3 (10·3–15·2) MJ) for boys. The median (IQR) daily nutrient intakes for girls and boys respectively were protein 64·6 (54·8–79·3) g, 74·4 (61·4; 89·4) g; carbohydrate 336·5 (285·3–393·6) g, 379·6 (317·8–461·8) g; and saturated fat 45·6 (34·8–58·3) g, 54·6 (41·9–69·5) g. There were no significant between-gender differences in percentage E from protein (10·2 (9·2–11·4)), or carbohydrate (52·4 (48·7–56·7)). Girls obtained less percentage E from saturated fat (16·1 (11·0–18·2) compared with boys 16·3 (14·2–19·1) (*P* < 0·05). E from saturated fat was above FAO recommendations in >74 % of participants. The estimated average requirement for iron was achieved by < 40 % of girls. In conclusion, strategies to optimise the dietary intake of adolescents in India should focus on preventing excess intakes of E and saturated fat and improving iron intake in girls.

India has the largest adolescent population (>253 million) in the world^(1)^. Early adolescence is a period of rapid development and growth^(2)^. Requirements of energy (E) peak as does the requirements for micronutrients, and therefore, adolescence is a period during which there is an increased risk of deficiency^(3)^. The results of the Comprehensive National Nutrition Survey (2016–18)^(4)^ show that a ‘triple burden’ of undernutrition, micronutrient deficiency^(5,6)^ and overnutrition exists in the young Indian population^(6–8)^. Dietary preferences and habits that form in adolescence may last lifelong, and therefore, it is an appropriate time to encourage and build healthy eating habits^(9)^.

The transition of the traditional Indian diet towards a more ‘westernised’ diet that includes prepackaged and processed food has been reported previously^(10,11)^. Existing food environments in lower-middle-income countries (LMIC) like India negatively impact dietary intake^(12)^. Children from the lower socio-economic status groups typically attend Government schools, and those from middle- and higher-income families attend private schools. Despite school feeding programmes actively operating in Government and Government-aided schools, children from lower income groups are disproportionately impacted by the unhealthy city food environments near schools with the offer and sale of processed food. On the other hand, children from middle- and high-income groups attending private schools have access to disposable income and are exposed to processed high-fat, sugar and salt foods within schools through cafeterias and through the location of outlets of multinational fast-food chains in the environment around school with no access to an organised school lunch programme^(13)^. Therefore, all the children and adolescents attending schools in Delhi, irrespective of the socio-economic status, are at risk of exposure to unhealthy processed foods, with the young adolescents attending private educational institutions facing a significant impact of consistent exposure within and outside schools. This is concerning as it contributes to a high risk of noncommunicable diseases^(13)^

The most recent available evidence shows that there is an increasing trend of the combined prevalence of childhood obesity and overweight in India with the highest reported combined prevalence from New Delhi^(4,14)^. This trend sustained in the latest (2019–21) report of the National Family Health Survey, which showed an increase in prevalence of overweight and obesity for men, women and children under 5 years compared with the previous survey year (2016). However, the prevalence data for obesity and overweight for the 11–14 years age group are not available in National Family Health Survey. The Comprehensive National Nutrition Survey data (2016–18) are the only available resource on prevalence of overweight (12·3 %) and obesity (3·6 %) for this age group^(4)^. Despite these concerning figures, there are no data on the individual dietary behaviour of this population.

Addressing undernutrition and ensuring food security remains the priority of the Government of India^(15)^. Food fortification and vitamin supplementation were implemented as stopgap measures to address micronutrient deficiency in vulnerable groups, i.e. low socio-economic status children and adolescent girls, specifically deficiencies of vitamin A, B12, folate, iron and iodine^(16)^. However, these approaches have been criticised for the lack of sustainability^(17)^. The results of haematologic investigations in the Comprehensive National Nutrition Survey 2016–18^(4)^ showed that iron deficiency anaemia was still prevalent in 40 % of adolescent girls in India^(18)^, and 24 % of adolescents were vitamin D deficient. The 2019–21 National Family Health Survey report has shown that more than half (51·5 %) of the adolescent girls in Delhi were anaemic, a further increase from the previous assessment in 2016.

The Government of India has now evolved its strategies to tackle the triple nutrition burden in adolescents through a package of interventions in the National Adolescent Health Program to be delivered in collaboration with other health agencies^(1,19)^. In addition to supply of fortified food in school lunches, approaches for adolescents to build an understanding of nutritious diet, make healthier choices and, avoid processed food are being implemented in Government schools but not in private schools. Policy-level action has been initiated to prohibit sale and marketing of unhealthy foods within and around schools^(20)^. There are, however, no high-quality data on the level of intake of nutrients including sugars from local foods, and the existing reports are limited to intake of food groups^(17)^ by adolescents. It is important to generate sub-national and national data on dietary intake to identify the level of any problem and inform the efforts to optimise eating behaviours.

Previous surveys of ten states of India of diet and nutrition status of rural^(21)^ and urban^(22)^ populations conducted in 2012 and 2017 respectively reported that the mean intakes of calcium, iron, folate, B12 and vitamin A in young adolescents did not meet the Recommended Dietary Allowance (RDA-the average daily dietary intake level that is sufficient to meet the nutrient requirement of nearly all (97–98 %) of healthy individuals). Since the publication of these reports, the reference values have been updated^(23)^ and estimated average requirement (EAR) values were defined by the National Institute of Nutrition (NIN). For a given nutrient, the EAR represents the nutrient intake value that is estimated to meet the requirement of 50 % of the healthy individuals. The NIN-EAR was suggested for the first time as the appropriate reference value for assessing requirements and adequacy in healthy individuals. Since the update of dietary reference values in 2020, no data on dietary behaviour have been collected and compared with the initial EAR for E and nutrients.

Research shows that lockdown during the COVID-19 pandemic had a significant impact on diet and physical activity^(24–27)^. In adolescents, compared with pre-pandemic period, screen time and intake of all food groups increased with a notable decline in physical activity^(28,29)^. In a study on young adolescents living in Mumbai, an increase in the intake of staple food and fruit, as well as foods high in sugar, fried food, sugar-sweetened beverages with a marked rise in snacking in between meals, was reported^(30)^. In Delhi, schools remained closed for two years due to the pandemic and reopened in 2022. In this post-pandemic context, an update on the nutrient intake would be useful to understand adolescent dietary behaviours.

Previous studies of dietary intake in India have relied on existing food composition databases and required researchers to manually analyse data^(17)^ for the estimation of intake of nutrients from reported food intakes, and these were usually collected using 24 h recalls or food frequency questionnaires^(21,22)^. However, there are no comprehensive local food and recipe databases. Further, the challenge posed by the lack of software to link the intakes to national food compositional tables has been identified^(17)^. Open-source and low-cost applications that facilitate assessment of dietary intake through the creation of a digital record of the food and drink intake are commonly used in high-income countries^(31,32)^. The application of such tools for dietary assessment in LMIC is limited or, as is the case with India, non-existent. The validity and reliability of one such tool ‘Intake24’ (https://intake24.org) has been tested^(32)^ and used in the national dietary surveys in other countries (e.g. the United Kingdom (UK) National Diet and Nutrition Survey)^(32–34)^. An English Language South Asia Locale (2022 version) of this application has recently been developed (https://intake24.co.uk/info/localisation) but it has not previously been applied to populations in India.

The aim of this study was to determine the intake of E and nutrients in 11–13-year-old young adolescents attending private schools in Delhi using the South Asia locale of Intake24. The specific objectives were to (i) estimate the intake of E and macronutrients and the contribution of macronutrients to E, (ii) to estimate the intake of vitamins A, D, B12, folate and iron, calcium, zinc, iodine and (iii) compare the intakes with the NIN-EAR.

## Methods

### Ethical approval

This study was conducted according to the guidelines laid down in the Declaration of Helsinki, and all procedures involving research study participants were approved by The Human Research Ethics Committee at The University of Adelaide (*H-2021-027*, Date: 8 March 2022) and by the Institutional Ethics Committee, Centre for Chronic Disease Control, New Delhi, India (*CCDC-IEC_14_2021* Date: 9 December 2021).

### Study location

Delhi is a Union Territory located in North India with a population of more than 16·7 million (2011 census)^(35)^. New Delhi, the capital city of India, is located within Delhi. There are eleven civic administrative districts and three municipalities, divided broadly into rural and urban sectors. The municipal corporations of North, East, South and New Delhi constitute the urban sector and villages (*n* 112) located in different districts constitute the rural sector.^(35)^.

### Sample size and school selection

Young adolescents in the age group of interest (11–13 years) (hereafter referred to as adolescents in this paper) attend middle schools in Delhi, i.e. sixth to eighth Grade. Since the primary aim of the doctoral project was to determine the data on the level of intake, principal dietary sources and patterns of free sugars intake in this sample of 11–13-year-old schoolchildren in India, the sample size calculations were based on the number of participants needed to estimate the total sugars intake in grams per day with a margin of error of ± 5 g/d.

The calculations assumed 80 % power to estimate mean total sugar intake and two-sided α of 0·05. The target sample size was set at 450 which was inflated to allow for 20 % attrition. It was anticipated that by recording the food and drink data from at least 360 adolescents, considering the total number of 11–13-year-olds in Delhi, we would be able to calculate the sugars intake data with a margin of error of ± 5 g/d. Private schools constituted 40 % of the total school enrolment in 2022 and 95 % of the 11–14 age demographic attended a school in Delhi. The number of private schools and students is not uniform across the eleven districts in Delhi. Therefore, using the complete list of 1374 private schools provided by the Directorate of Education, Government of National Capital Territory Delhi, a statistician external to the research team generated a schedule containing the names of 150 random schools, stratified by district from across the eleven districts of Delhi. Using this schedule, schools were invited to participate, and recruitment continued until ten schools had consented.

### School consent

Using the list of 150 schools, a researcher (AI) contacted the principal of each school through email and on telephone and explained the purpose of the study in plain language. The researcher made three attempts to connect with the school and explain the study information. If no response was obtained or if the school declined to take part, the researcher moved to the next school on the list for that specific district. This process was repeated until the researcher recruited at least one school from each district. Researchers collaborated with Health-Related Information Dissemination Amongst Youth, a nongovernmental organisation based in Delhi, for establishing initial contact with the principals of private schools. The researcher shared information about the study with those schools that expressed interest to take part in the research through email. Once a school agreed, a link to the school consent form was sent by email (Qualtrics XM Experience Management, North Sydney, Australia). Upon obtaining consent, the process for recruitment of participants was initiated.

### Parent consent and participant assent

A template letter for inviting parents of the 11–13-year-old students to take part in the study was prepared by the researcher (AI) and provided to the school. Within each school, a dedicated teacher provided study information (prepared in plain language in both Hindi and English by the researcher AI) to the parents of the 11–13-year-old students through the school network on social media applications (WhatsApp) or through emails. Parents of interested students communicated their willingness to participate to the teachers. The teacher then created a list of all interested participants. Next, the teacher asked the parents to fill in and sign the online parent consent form by sharing a link to this form (hosted on Qualtrics) through the school’s social media network. Some schools requested the researcher to provide a brief orientation about the research to the group of participants who were selected by the school. This talk was organised by the school and held in the school’s auditorium or in a classroom. During this interaction, the researcher explained in detail the purpose of the study and the expected level of involvement of the participants. The researcher requested participants to share the study information with parents. Only after the researcher received a filled-in and signed consent form (made available in English and Hindi) from the parent(s), the student was recruited as a participant in the study. A participant information sheet was shared by the researcher for the participants to further understand the purpose of the study and the level of their involvement. Participants provided assent online (hosted on Qualtrics) before data collection commenced. The numbers of participating students from each school varied. This was based on the total number of 11–13-year-old students in the school, and the number of participants for whom parental consent has been obtained.

### Collection of demographic data

Demographic data on participants were collected from their parents using an online questionnaire, using the Qualtrics survey tool. The variables included name, age and date of birth of the child, name and age of the parent and the area of residence. As part of this questionnaire, parents were asked to record at home or provide from previous records, the weight (in kilograms) and height (in inches or centimetres) of their child.

### Dietary data collection

Dietary data were collected between May and October 2022. Participants recorded all foods and drinks consumed over three consecutive days in paper-based or online version of a purpose-designed food diary. The instructions for filling in the diary were provided in English and Hindi. Recording included two weekdays and one weekend day. This allowed estimation of the dietary intake during school days (Friday, Saturday, Monday and Tuesday) and non-school days (Sunday). Participants were asked to report all food and drink consumed including the time of consumption and the amount that was consumed using household measures (cups, bowls, spoons, glasses and plates etc.) After completing the diary, the researcher met each participant in a one-on-one online interview to discuss, clarify and enlarge on the information provided and to confirm the portion size of foods and drinks consumed using the library of 2500 food portion sizes available in the online dietary assessment tool Intake24 (South Asia Locale 2022 version)^(32,33)^. During the interview, the researcher entered the dietary information into Intake24 which enabled the daily intake of E and sugars to be derived through integrated food composition tables. The Intake24 tool captures data on nutrient supplementation. However, these data were not analysed in this study as the intake of nutrients from foods was of interest.

Following the completion of all interviews, the resulting data file was downloaded as an Excel spreadsheet from Intake24. The dietary data were imported into STATA 17·0 (Stata Statistical Software: Release 17. College Station, TX: Stata Corp LLC). In the dataset, the daily dietary data for each participant for each of the three days were organised into eating occasions. The pre-defined eating occasions in Intake24 included the following: (1) early snack or drink; (2) breakfast; (3) morning snack or drink; (4) lunch; (5) afternoon snack or drink; (6) evening meal and (7) late snack or drink. Water intake was recorded at the end of each day as daily water intake. In the spreadsheet, the data were identified through user ID which included name of the school and the numeric participant ID (1 to n for each school). Indexing variables were created for the school, the participant, the day and the eating occasion. The total intake of E (megajoules), grams of carbohydrates, protein, fat, saturated fat and fibre and vitamins A, B12, folate and D, and minerals iron, calcium, zinc, iodine and selenium for each of the three days of recording was derived and then averaged to get the daily intake of E and nutrients for each participant.

### Validation of energy intake

The estimation of physical activity level (PAL) was used to validate the reported E and identify the proportion of participants who may have over- or under-reported E. The PAL was calculated by dividing the reported E by the basal metabolic rate. Schofield equation^(36)^ was used to predict the basal metabolic rate from gender, age, height and weight information provided by the participants.

### Statistical analyses

The distribution of variables was assessed. Descriptive statistics for continuous variables that were normally distributed included mean, SD and 95 % CI and for variables with non-Gaussian distribution median and interquartile range (IQR) were reported. Between gender differences for E, macronutrient and micronutrient intake and percentage E obtained from macronutrients were explored through Wilcoxon rank-sum tests. A two-sided significance level of 0·05 was used in all analyses.

## Results

### Participation rate

**Fig. 1.**
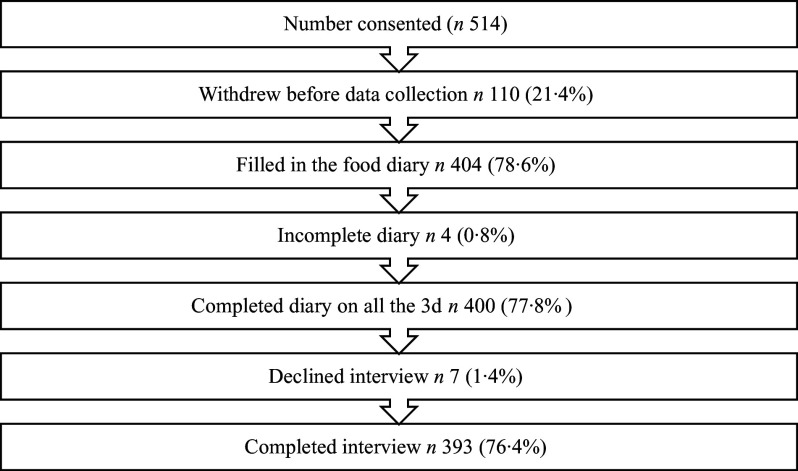
Participation rate from ten private schools in Delhi, India.

During the school recruitment process, twenty-seven schools declined to participate, and fifteen schools could not be contacted. In total, fifty-two schools were approached before the required sample of ten was achieved. Between June and October 2022, students from each school consented. Of the 514 participants in total who provided consent, 393 (169 girls, 224 boys) completed the diary on all three days and attended the interview (76·4 %) (Fig. 1). The mean age of the participants was 11·4 (sd 1·8) years.

### Energy intake and physical activity level ratios

The distribution of the E, macronutrient and micronutrient intake data was skewed, and therefore, the data have been summarised as medians and IQR. The median IQR daily E intake was 2580 (2139·3–2989·8) kcal (10·8 (9·0 − 12·5) MJ) for girls and 2941·5 (2466·7–3599·3) kcal (12·3 (10·3–15·2) MJ) for boys (Table 1). The PAL ratios derived for those 241 participants who provided height and weight were 2·0 for girls and 2·1 for boys. The median weight of the ninety-seven (57·4 %) girls who provided anthropometric data was forty-five (IQR 38; 51) kg and of the 144 (67·3 %) boys who provided data was 44·1 (IQR 37; 52·5) kg. The median weight for the study population was above the reference weight for Indian 10 to 12 years old (34·9 kg for boys and 36·4 kg for girls) ^(23)^.

**Table 1.** Energy, macronutrient intake and percentage contributions of macronutrients to energy for 393 11–13-year-old adolescents (224 boys and 169 girls) attending private schools in Delhi, India (Median values and IQR values)

	All participants		Boys (*n* 224)				Girls (*n* 169)			
Variable	Median	IQR	Median	IQR	% > EAR	NIN-EAR	Median	IQR	% > EAR	NIN-EAR
E intake										
MJ/day	11·6	9·9–14·1	12·3	10·3–15·2	80·4	2220 kcal	10·8	8·9–12·5	81·7	2060 kcal
kcal/day	2761	2348·4–3358·1	2941·5	2466·7–3599·3			2580	2139·3–2989·8		
Carbohydrate										
grams/day	360·2	300·1–441·9	379·6	317·8–461·8	99·6	100 g/d	336·5	285·3–393·6	99·4	100 g/d
% E	52·4	48·7–56·7	51·9	48·4–56·3			52·9	49·5–57·5		
Protein										
grams/day	69·6	58·6–85·8	74·4	61·4–89·4		26·2 g/d	64·6	54·8–79·3		26·6 g/d
% E	10·2	9·2–11·4	10·1	9·2–11·4	52·7		10·4	9·2–11·5	53·8	
Fat										
grams/day	124·7	98·9–155·6	128·9	106·5–165·6		35 g/d	114·1	90·3–138·9		45 g/d
% E	40·4	36·1–44·2	40·8	36·7–44·5	74·1		40·1	35·1–43·3	65·1	
Saturated Fat										
grams/day	51·6	37·1–63·0	54·6	41·9–69·5		–	45·6	34·9–58·3		–
% E	16·3	13·9–18·5	16·3	14·2–19·1	90·2		16·1	11·0–18·2	90·5	

IQR, interquartile range; EAR, estimated average requirement; NIN, National Institute of Nutrition

### Macronutrient intakes

The information on the median daily intakes of carbohydrate, fat, saturated fat and protein is detailed in Table 1. There were no significant between-gender differences in percentage E from protein: median 10·2 (IQR 9·2–11·4); boys 10·1 (9·2–11·4) *v*. girls 10·4 (IQR 9·2–11·5), or carbohydrate: median 52·4 (IQR 48·7–56·7); boys 51·9 (48·4–56·3) *v*. girls 52·9 (49·5–57·5). A comparison of the macronutrient intake (g) of the study population with NIN-EAR for Indian 10–12-year-olds^(23)^ and the percentage E contributed by macronutrients with the FAO recommended ranges^(37)^ is also presented in Table 1. The absolute intakes of macronutrients (g/d) were above the India NIN-EAR for 10–12-year-old population. The percentage E contributed by carbohydrate and protein was within the recommended FAO ranges but the contribution of saturated fat to E was considerably above the recommended threshold of no more than 10 %^(37)^. Girls obtained less percentage E from saturated fat (16·1 % (IQR 11·0–18·2) compared with boys 16·3 % (IQR 14·2–19·1) (*P* < 0·05).

### Micronutrient intakes

The median intakes of micronutrients are summarised in Table 2, along with the percentage of participants achieving NIN-EAR for the intake of micronutrients.

**Table 2.** Micronutrient intake by 393 11–13-year-old adolescents (224 boys and 169 girls) attending private schools in Delhi, India (Median values and IQR values)

	All participants		Boy (*n* 224)				Girls (*n* 169)			
Variable	Median	IQR	Median	IQR	% achieving EAR	NIN-EAR	Median	IQR	% achieving EAR	NIN-EAR
Vitamin A (µg)	1082·9	839·4–1346·4	1132·9	918·1–1406·4	96·4	360	953·2	767·7–1246·6	94·7	370
Vitamin D (µg)	1·7	0·9–3·6	1·9	1·0–4·0	7·1	10	1·4	0·7–3·0	4·1	10
Vitamin B12 (µg)	3·2	1·8–4·6	3·3	2·0–5·0	73·2	2	2·9	1·6–4·2	63·3	2
Folate (µg)	261·5	204·0–315·3	267·8	205·7–321·7	72·8	180	253·6	203·0–303·8	72·2	186
Calcium (mg)	1105·1	797·4–1551·7	1183	836·6–1646·3	84·4	650	1033·5	778·6–1406·7	75·7	650
Iron (mg)	15·1	12·4–18·4	15·7	13·3–19·0	72·8	12	14	11·6–17·0	37·9	16
Zinc (mg)	9·4	7·8–11·8	9·9	8·2–12·7	81·7	7·0	8·6	7·3–10·6	72·2	7·1
Iodine (µg)	157	108·7–216·6	168·6	116·1–223·4	78·6	100	142·5	102·8–209·8	72·8	100
Selenium (µg)	34·2	27·2–43·0	35·9	28·4–44·1	37·1	40 (RDA)	31·6	26·7–41·7	30·2	40 (RDA)

IQR, interquartile range; EAR, estimated average requirement; NIN, National Institute of Nutrition, RDA Recommended Dietary Allowance.

Nearly all the participants (95 %) achieved the EAR for daily vitamin A intake. In contrast, only 7 % of boys and 4 % of girls achieved the NIN-EAR for Vitamin D. The median daily intakes of Iodine in girls iron, selenium and vitamin D in both girls and boys were below the NIN-EAR. Less than 40 % of girls achieved the NIN-EAR for daily iron intake.

## Discussion

In this sample of adolescents living in Delhi, the intakes (g/d) of protein, carbohydrate and fat were above the NIN-EAR. The median E intake 2761 (2348·4–3358·1) Kcal/d (11·6 (9·9–14·1) MJ/d) was higher than that previously reported for similar aged adolescents living in some high-income countries in the WHO European^(38)^, American^(3,39)^, Eastern Mediterranean^(40)^, Western Pacific Regions^(41)^ and LMIC like Libya^(42)^ but is similar to intakes reported for adolescents in the African and South East Asian regions; e.g. 9·7 MJ/d in Cameroon^(43)^ and approximately 12·3 MJ/d in Bangladesh^(44)^. The E intake in the current study was validated against the estimation of PAL using data provided by a sub-sample (*n* 241). The PAL for this sub-sample falls in the ‘vigorous activity’ category of the FAO classification; however, the higher than desirable median weight (45 kgs) and high median E intake is suggestive of overeating. Hence, there is a need to control E intake in order to prevent obesity. However, it is possible that participants may have overestimated portion sizes for social desirability. Moreover, Intake24 sometimes relies on best-match foods from the UK food compositional database when data on the composition of local foods are unavailable. Further development of the SE Asian version of Intake24 to include more specific compositional data is desirable for future surveys.

### Macronutrient intake

Of concern is the percentage contribution of saturated fat to E (median 16·3 %), which is above the 10 % E threshold for intake of saturated fats recommended by the WHO FAO and other authoritative agencies^(23,37)^. More than 90 % of the current study sample consumed above 10 % E as saturated fat and dietary fat contributed more than 35 % to total E in 75 % of the boys and 65 % of girls. This figure is higher than the reported contribution of saturated fat to E in the diets of 11–18-year-olds living in the UK (median 12·6 %)^(45)^ and 13–18-year-olds in Europe (14·0 %)^(38)^ and the Middle East (15·0 %)^(40)^. Due to the known association between a diet high in saturated fat and risk for noncommunicable diseases^(46)^, strategies to lower the intake of saturated fat to less than 10 % E in adolescents need consideration. The intake of the ready-to-eat and prepackaged foods reported by the current study sample as ‘snacks’ probably contributed to the high-fat intake^(47)^. Such foods are widely available within private schools in the cafeteria and in school food environments^(48)^. It is important to find means and strategies to support and promote the intake of nutrient-dense food and reduce the consumption of high-fat energy-dense food.

The percentage E from carbohydrates and fat in the current study was lower than that in diets of adolescents in the UK^(45)^ and many high-income countries in Europe^(38)^ and Middle East^(40)^ and in LMIC such as Lebanon, Libya and Morocco^(40)^. The balance of carbohydrate, fat, saturated fat and protein in the diets of adolescents should be investigated further to inform strategies that promote healthy eating by this population. The intake of energy-dense and nutrient-poor prepackaged snacks especially by schoolchildren is driven by the ubiquitous sale and marketing of such foods. A lack of policy in India that calls for limiting the use of saturated fats in the manufacture or preparation of snack foods compounds the scenario around snack food intake.

The median percentage E contributed by protein in the current study sample (10 %) is lower than the contribution of protein (approximately 15 %) in the diets of adolescents aged between 11 and 18 years in the UK^(45)^ and between 13 and 18 years in Europe^(49)^ and Middle East^(40)^ but is similar to the reported intake of protein for similar aged adolescents attending schools in LMIC, for instance Cameroon (10 %)^(43)^ and Bangladesh (9 %)^(44)^. The 2017 survey on urban populations in India has reported that the intake of protein is low when compared with the recommended dietary allowance^(22)^. Further, results from a survey that compared Indian diet with the Lancet EAT-reference diet indicate that the intake of plant and animal-based protein is low across all sectors, regions and income groups in India^(50)^. The sources of protein, saturated fat and carbohydrates in the diet of this population should be investigated further to inform strategies to promote a balanced diet. While large sections of the populations consume a carbohydrate-based vegetarian diet during the week and limit intake of meat-based protein sources to weekends and special occasions, it would be useful to investigate the factors that drive the protein intake patterns of adolescents to improve intake.

### Micronutrient intake

The intake of micronutrients was compared with the NIN-EAR, except for selenium. The India NIN provides the recommended dietary allowance for daily selenium intake but no EAR. Median intakes below the NIN-EAR were observed for the intake of vitamin D, folate, iron and zinc.

The finding that the intake of Vitamin A was above the NIN-EAR in nearly all participants (95 %) contrasts with the findings for school-age adolescents in developing countries which have reported the status of Vitamin A to be inadequate in > 85 % of adolescents in Ethiopia and > 80 % of adolescents in Cameroon and Uganda^(51)^. The current study figure is also unlike the 2012 and 2017 survey findings^(21)^ which showed that vitamin A intake was inadequate in 52–85 % of adolescents. However, these previous reports compared the intake with the recommended dietary allowance for vitamin A (600 µg/d). In addition to the difference in methodology to report the adequacy of intake, it is also likely that the results are different due to the reported change in diet with a shift towards intake of raw vegetables and in some participants, supplementation.

The low intake of Vitamin D in the current study population is similar to that reported for adolescents in UK (1·8 µg/d)^(45)^. Dietary sources of Vitamin D are few; however, it is likely that the study population benefited from subcutaneous synthesis of vitamin D. According to the serologic investigations conducted as a part of the 2016–18 Comprehensive National Nutrition Survey,^(4)^ nearly half of the (47·1 %) adolescents in Delhi were found to be deficient in Vitamin D. Information of the serum concentrations of 25(OH)D (25-hydroxy cholecalciferol) would provide more comprehensive information on Vitamin D status. Possible changes in the exposure to sunlight due to the confinement of participants indoors because of the pandemic may have affected the vitamin D status.

The median daily intake of folate by girls in the present study (254 µg/d (IQR 203–304)) is considerably higher than the previously reported intake (165 µg/d) for south Indian adolescent girls^(52)^. Of concern, however, are the relatively low intakes of iron by girls found in the current study. Inadequate status of iron in adolescent girls has previously been reported for several developing countries in Africa, Southeast Asia and Middle East^(51,53)^. More than 60 % of the girls in the current study did not achieve the NIN-EAR for iron intake. The figure is much higher with 73 % of boys achieving the NIN-EAR. Information on menarche was not collected in the current study which may have provided clarity on the demand for iron and folate in girls. Both the 2012 and 2017 surveys in India^(21)^ showed that adolescent girls and boys are unable to achieve adequate iron intake. Girls have poorer dietary intake in general compared with boys. The information on the sources of iron in food and the pattern of intake of iron by girls and boys could be investigated further to understand the factors that inform the differences in percentage of boys and girls achieving the NIN-EAR for Iron.

Furthermore, more than half of anaemia cases are estimated to be due to iron deficiency^(54)^. In India, anaemia is a recognised public health problem. One priority of the National Adolescent Health Program^(19)^ is weekly micronutrient supplementation through the provision of iron and folic acid tablets. However, only adolescents in government, government-aided and municipal schools are covered^(7,19)^. The current study results suggest the need to improve iron intake by girls. The fact that the current study sample of adolescents attending private schools does not have access to the fortified food supply through school lunches suggests that there is a need to explore the feasibility of expanding the weekly supplementation to adolescent girls in all schools.

Compared with intakes reported for adolescents in previous studies conducted in LMIC in Africa^(51,53)^ and Middle East^(51)^, median zinc intake (8·6 mg/d) was high with 72 % of girls achieving the NIN-EAR. Participants in the present study reported intake of zinc-containing foods as a part of main meals, e.g. *dal*, and pulses like chickpeas, soybeans, rajma beans, etc., which may have contributed to adequate zinc intake. Compared with previously reported intake for adolescent girls in India,^(52,55,56)^ the zinc intake is high.

The proportion of the study population which achieved the NIN-EAR for iodine (75 %) is higher compared with previous estimates^(57)^. Salt fortification with iodine was a crucial component of a national programme that aimed to prevent and control the endemic iodine deficiency disorders in India which also lead to a ban on the sale of non-iodised salt. Although information on the intake of iodised salt was not specifically obtained in the current study, this is likely to have contributed to adequacy of iodine intake. Thirty percentage of girls and 37 % of boys achieved the NIN-recommended dietary allowance for selenium intake (40 µg/d, EAR not defined). There are few comparative data on selenium intake available although the intake was comparable with that reported for UK adolescents^(45)^. However, recently available data from the estimation of serum selenium levels showed that 10 % of urban young adolescents in India and a majority of adolescents in LMIC in Africa have inadequate serum selenium^(55,58)^.

### Strengths and limitations

Revised nutrient recommendations for intake of E and nutrients in the Indian population at different ages were proposed in the year 2020. To the authors’ knowledge, this is the first assessment of dietary intake reported since then. The data on nutrient intake are the first to be collected using the Intake24 South Asia Locale and the results provide an update on the status of nutrient intake in schoolchildren since the pandemic. This research study serves as an example for application of a validated digital tool for assessment of nutrient intake in school settings and provides the opportunity to discuss the scope of further research through localisation of the tool for dietary surveys in India.

Nonetheless, there are some limitations which need to be acknowledged. First, participant-reported and not researcher-measured height and weight were used to derive PAL. The height and weight data were available for only 61·3 % of the sample which may have impacted the accuracy of the PAL ratios. Second, Intake24 is designed to be a self-completed multiple pass 24 h recall dietary analysis tool. However, the South Asia locale is available only in the English language which was not the first language for many participants. Therefore, the food and drink intake data were collected using a 3d dietary diary and the researcher transferred this information to Intake24 which was used to ascertain the portion size (during a participant-researcher interview) and to translate recorded food intake into the intake of nutrients. Although the participants did not directly enter dietary data, the library of portion size photographs enabled portion size estimation. Third, despite Intake24 South Asia locale being the most comprehensive dietary assessment tool designed to be used in all the Southeast Asian countries, the nutrient composition of every Southeast Asian food and drink item is not available. Therefore, the system finds a best match from the UK food composition tables which may introduce some inaccuracies. Fourth, the study was conducted between May and December 2022, a time during which participants were transitioning back into in-person learning at schools after the COVID-19 lockdown led to school closures. The changes to diet reported by participants, i.e. increase in the intake of raw vegetables, citrus fruit juices and whole fruits^(25,27)^ which persisted even after schools reopened, could have influenced the nutrient intakes. Finally, it is possible that the data were subject to bias of social desirability so that unhealthy food or drink items may have been under-reported, and participants may have overestimated the portion sizes during the interview with the researcher^(59,60)^.

### Future directions

The results show that it is possible to obtain individual and population-level data including the daily intake of E and nutrients through the use of Intake24. The capture of data using Intake24 reduced research burden by translating reported food intake into daily intake of nutrients. Intake24 should be further developed into local languages and expanded to include a more comprehensive coverage of foods and dishes that are part of the food culture of India.

The findings of this study pertaining to the intake of E and macronutrients suggest that interventions including policy measures are needed to prevent excess E intake provided by fat, especially saturated fat. The results relating to the intake of iron in girls suggest targeted strategies to reduce the risk of anaemia in adolescence by improving dietary intake through measures such as food fortification may be required. Further research should be undertaken to understand the balance of fat intakes and patterns of protein intake by adolescents in India to underpin the strategies to lower exposure of schoolchildren to deep-fried snack foods and encourage healthy eating.

### Conclusion

The E intake in this sample of young adolescents attending private schools in Delhi, India, was above the level recommended by FAO and the India NIN-EAR. The intake of saturated fat was above the <10 % E threshold, with higher intakes in boys compared with girls. The majority of the girls did not achieve the NIN-EAR for iron. Strategies to optimise dietary behaviours in young adolescents attending private schools in India should focus on preventing excess energy intakes, reducing the intake of saturated fat and promoting increased intake of iron in girls.
